# Adapting to heatwave-induced seagrass loss: Prioritizing management areas through environmental sensitivity mapping

**DOI:** 10.1016/j.ecss.2022.107857

**Published:** 2022-08-05

**Authors:** Sara Pruckner, Jacob Bedford, Leo Murphy, Joseph A. Turner, Juliet Mills

**Affiliations:** a UN Environment Programme World Conservation Monitoring Centre (UNEP-WCMC), 219 Huntingdon Road, CB30DL, Cambridge, United Kingdom; b Fauna & Flora International, The David Attenborough Building, Pembroke St, Cambridge, CB2 3QZ, United Kingdom

**Keywords:** Seagrass, Marine heatwaves, Mediterranean, Fisheries, Coastal hazards, Coastal erosion, Posidonia oceanica, Climate change, Ecosystem services, Environmental sensitivity mapping

## Abstract

Seagrass meadows support complex species assemblages and provide ecosystem services with a multitude of socio-economic benefits. However, they are sensitive to anthropogenic pressures such as coastal development, agricultural run-off, and overfishing. The increasing prevalence of marine heatwaves (MHWs) due to climate change poses an additional and growing threat. In this study, we apply the environmental sensitivity mapping approach MESA (Mapping Environmentally Sensitive Assets) to explore the potential consequences of MHWs on the ecosystem services that *Posidonia oceanica* provides to coastal communities. Under the intermediate climate change scenario Representative Concentration Pathway 4.5, Mediterranean marine heatwaves will be severe by 2050, and will very likely increase mortality of *P. oceanica*. However, the societal risk of seagrass loss is not evenly distributed across the Mediterranean. The spatial distribution of socio-economic implications of seagrass loss is highlighted through two case studies on seagrass-dependent fisheries and coastal hazards. Coastal communities in Tunisia and Libya show very high sensitivity to a loss of fisheries due to a combination of increasingly intense and frequent MHWs, coupled with high proportions of regional seagrass-dependent fisheries catch. The coastlines of Italy, Tunisia, and Cyprus are shown to potentially be highly sensitive to loss of seagrass due to high levels of coastal hazards, and seagrass meadows susceptible to MHW-induced degradation. These coastlines are likely to suffer from reduced coastal protection services provided by intact seagrass meadows. We demonstrate the implications of MHWs for ecosystem service provision to coastal communities in the Mediterranean and the need for policy instruments to help mitigate and adapt to its effect. We also highlight the potential for environmental sensitivity mapping to help support policymakers with rapid screening tools to prioritize resources more effectively to areas where in-depth local planning is needed.

## Highlights

• *Posidonia oceanica* provides important ecosystem services for coastal communities.
• *P. oceanica* is affected by magnifying marine heatwaves due to climate change.
• MHWs will affect the provision of fishing and coastal protection services.
• Areas that heavily rely on these services should prioritize adaptation planning.
• Environmental Sensitivity Mapping is used here to prioritize adaptation responses.

## Graphical abstract

**Figure:**
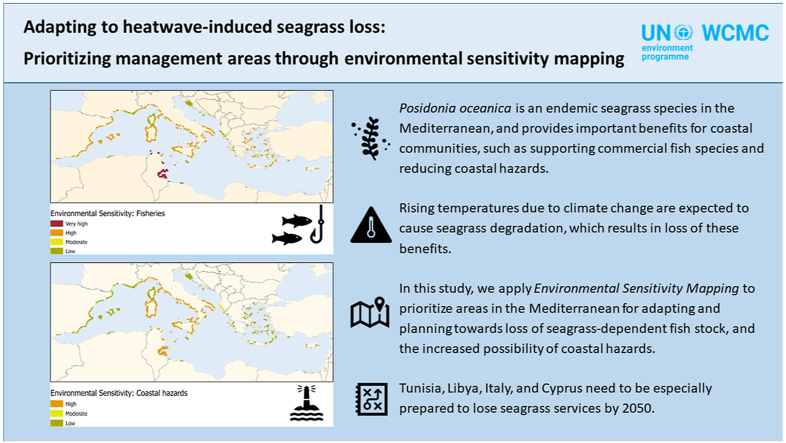

## 1 Introduction

The importance of the Mediterranean Sea for people and nature has been widely documented. Its extensive coastal wetlands, including 1.2 million hectares of seagrass meadows, support 68 endemic and at least 78 globally threatened species, and underpin many local economic activities such as those based on tourism and fishing (United Nations Environment Programme/Mediterranean Action Plan and Plan Bleu, 2020).

However, Mediterranean seagrass meadows and the services they provide are threatened by a wide range of pressures, such as coastal development and poor water quality. Exacerbating these direct anthropogenic pressures is the growing threat from climate change, including the increased occurrence of marine heatwaves (MHWs) (Jordà et al., 2012). Marine heatwaves are defined as periods of extreme warm sea surface temperature (SST) that persist for days to months and vary in extent (Oliver et al., 2021). The occurrence of MHWs is increasing globally, and their frequency and intensity is projected to continue growing under climate change (Oliver et al., 2018). The Mediterranean Sea is projected to be particularly affected by climate change (Giorgi, 2006) which is expected to cause an increase in MHW frequency and intensity, with both the mean and the variability in sea surface temperatures likely to be affected (Darmaraki et al., 2019).

The endemic species Posidonia oceanica, which forms the majority of Mediterranean seagrass meadows, is particularly threatened by increasing sea temperatures and could potentially lose up to 75% of its suitable habitat by 2050, and become functionally extinct by 2100 (Chefaoui et al., 2018). MHWs have been shown to be a primary mechanism through which climate change can affect *P. oceanica*. For example, the highest Sea Surface Temperature (SST) of a heatwave has been shown to be a key predictor of *P*. *oceanica* decline (Marbà and Duarte, 2010). Intense heatwave exposure can negatively affect growth rates, health, and mortality of seagrass, with younger life stages being particularly impacted (Díaz-Almela et al., 2009; Hendriks et al., 2017; Jordà et al., 2012; Marbà and Duarte, 2010; Olsen et al., 2012).

Given the economic importance of seagrass to the Mediterranean region, declines in seagrass extent and condition due to heatwaves are a societal risk which must be assessed and planned for. Environmental sensitivity mapping (ESM) is one method that combines environmental spatial datasets for an assessment of the sensitivity of an environmental asset, such as a habitat or ecosystem service, with the broader goal of informing planning processes (González Del Campo, 2017). The resulting environmental sensitivity maps display the relative sensitivity of the chosen asset to a given pressure, helping regulators to identify areas that should be prioritized for restoration, conservation, and sustainable management activities.

Here we apply an established ESM approach called “Mapping Environmentally Sensitive Assets” (MESA) (NEA and UNEP-WCMC, 2020) to identify areas of the Mediterranean that are sensitive to a reduction of ecosystem service provision as a result of the degradation of *P. oceanica* around 2050 due to the impact of marine heatwaves. The approach was selected as it provides a rapid, repeatable protocol that can easily be conducted by decision-makers to provide high level screening of locations requiring more in-depth attention and resources. This approach has successfully been tested in the low-resource context of developing countries, requiring only limited technical capacity and computational power. The goal is to help decision-makers understand where sensitive areas are located, and help to plan future actions to mitigate and adapt to the environmental and social risks associated with different pressures, such as climate change.

## 2 Methods

**Fig. 1:**
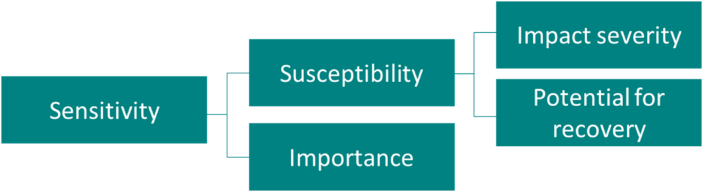
Conceptual process behind the MESA approach to sensitivity assessments (adapted from NEA and UNEP-WCMC, 2020).

The MESA approach (Fig. 1) defines *sensitivity* of an ecological asset as a function of the *importance* of an area for providing ecosystem services, and the *susceptibility* of the area to degradation as a result of a pressure. In this study, susceptibility is the degree to which a seagrass meadow will be affected by a marine heatwave, based on the predicted severity of the impact and the meadows' ability to recover once the pressure has ceased. Importance is, in this study, based on a broad indication of the relative value of a seagrass meadow in relation to its role in providing mitigation of coastal hazards, in particular coastal erosion and the reduction of wave height and speed, and supporting fisheries.

### 2.1 Calculating susceptibility

The susceptibility of *P. oceanica* to MHW-induced degradation is associated with both the intensity (Marín-Guirao et al., 2016; Traboni et al., 2018) and duration of the MHW (Olsen et al., 2012), as well as their frequency (Guerrero-Meseguer et al., 2017) and the intervals between them (Ruiz et al., 2017).

We apply MESA's categorical approach to scoring for the two components of susceptibility—impact severity and potential for recovery—assigning values between 1 (very low susceptibility) and 5 (very high susceptibility) for each. Each seagrass patch was assessed independently, based on the conditions it will be exposed to during the study period 2046–2055, averaging around the year 2050. We define impact severity based on the intensity and duration of MHWs. For this we use the maximum SST and maximum heatwave duration, with each factor given equal weighting (Table 1). The potential for recovery is defined by determining the number of heatwaves in a season, and number of days between heatwaves at SST values that do not pose heat stress to *P. oceanica*. For both impact severity and potential for recovery, the mean score across both factors is used.

**Table 1:** Scores assigned depending on thresholds of impact severity and potential for recovery.

Susceptibility score	Impact Severity		Potential for recovery	
	Max SST	Max. MHW length	No. of HWs in a season	Time between heatwaves
**1 (Very low)**	<24 °C	≤4 days	0	≥60 days
**2 (Low)**	≥24 °C <27 °C	5–10 days	1	40–59 days
**3 (Moderate)**	≥27 °C <29 °C	11–20 days	2	20–39 days
**4 (High)**	≥29 °C <32 °C	20–30 days	3	5–19 days
**5 (Very high)**	≥32 °C	≥31 days	≥4	<5 days

**Maximum SST:** Sea surface temperatures of 24 °C are regarded as baseline temperatures that do not present any heat stress to *P. oceanica* (Marín-Guirao et al., 2016; Traboni et al., 2018). While this threshold is likely to be higher in the Eastern and Southern Mediterranean, this paper is taking a precautionary approach, selecting temperatures at the lower end for the purposes of a high-level screening across the entire Mediterranean. Where sensitivity is identified, more detailed local assessments can be carried out, which may adjust the temperature thresholds based on increased tolerance. Temperatures below this threshold were therefore considered to present the lowest possible impact for seagrass. Jordà et al. (2012) and Marbà and Duarte (2010) investigated the relationship between shoot mortality of *P. oceanica* in the Mediterranean and the maximum annual SST. They established a linear relationship between the single highest SST observed in a year and shoot mortality in that year. According to this relationship, a maximum SST of 32 °C would lead to 20% shoot mortality each year. This is detrimental considering the low recruitment rates of 0.02–0.5 per year (Marbà et al., 1996). We therefore assign SST equal to or above 32 °C as the highest possible impact severity. Despite regional differences in the ability of *P. oceanica* to withstand high SSTs within the Mediterranean, this cut-off point is highly likely to cause severe damage to all varieties, so a precautionary approach of a lower threshold was taken. In between these extremes, impact rankings (Table 1) are based on the results of multiple experiments and studies, where growth was found to be noticeably impaired above 27 °C, and even more so at 29 °C and above (Buñuel et al., 2021; Guerrero-Meseguer et al., 2017).

**Maximum MHW duration:** The impacts of heat stress have been shown to be more severe with increasing duration of MHWs (Olsen et al., 2012), with a range of studies exploring different durations, including a single high temperature event (Díaz-Almela et al., 2009; Jordà et al., 2012; Marbà and Duarte, 2010), heatwaves lasting five days (Marín-Guirao et al., 2016), 7–21 days (Hendriks et al., 2017), 28 days (Traboni et al., 2018) and longer than a month (Buñuel et al., 2021; Guerrero-Meseguer et al., 2017; Olsen et al., 2012). The above reviewed studies showed that the longer a heatwave lasts, and therefore the longer *P. oceanica* is exposed to high temperatures that pose heat stress, the more affected growth, extent, and survival of seedlings and shoots will be. There is not enough data to identify firm thresholds and boundaries between values. However, no study showed significant effects of heat on *P. oceanica* for a heatwave duration of less than 5 days, whereas all studies reviewed observed significant stress if temperatures lasted for a month or more. As a result, we selected these as the boundaries for categorisation (Table 1).

**Marine heatwave frequency:** The frequency of MHWs is an important factor in determining the recovery potential of *P. oceanica* after marine heatwaves, with each MHW increasing the potential damage (Aoki et al., 2021; Perkins et al., 2012). More frequent marine heatwaves mean more significant damage to a seagrass meadow, less opportunity for recovery, and increased mortality (Guerrero-Meseguer et al., 2017). Seagrass meadows that experience no marine heatwaves in any one season will of course have no susceptibility to harm; while every single additional heatwave has an increased potential impact on the seagrass meadow, resulting in a linear increase in scores with every additional heatwave for this study.

**Recovery time between:**Marbà and Duarte (2010) state that time between marine heatwaves is a crucial factor in determining the survival of a *Posidonia oceanica* meadow. However, not a lot of studies exist that provide clear data on the time necessary for recovery between heatwaves. When a seagrass meadow has fully died, it will often not regrow naturally, or only start to regrow after several years, depending on factors influencing recolonization (Gera et al., 2014; Guerrero-Meseguer et al., 2017). In these very significant cases, risk of losing a seagrass meadow would be so high that likelihood of recovery could be disregarded when thinking about short- or medium-term socio-economic risk. In cases of only partial mortality, *P. oceanica* has a low recovery rate due to a slow natural growth rate, with a recovery rate of 7% per year without further disturbances, depending on the severity of impact (Gera et al., 2014). Laboratory experiments concluded that after a heatwave of 5 days at 32 °C, plants recovered after 5 days at 24 °C (Marín-Guirao et al., 2016), while other studies observing the effects of 6 weeks at just 27 °C, found that after 6 weeks of 23 °C, leaf growth had also recovered (Ruiz et al., 2017). We assume here that while the relationship is complex, the longer the time between a heatwave at temperatures that do not pose heat stress, the more *P. oceanica* can recover before a new heatwave. For this study, we therefore chose thresholds between 5 and 60 days that might be sufficient for partial recovery, depending on other factors such as max SST.

#### 2.1.1 Data analysis

**Fig. 2:**
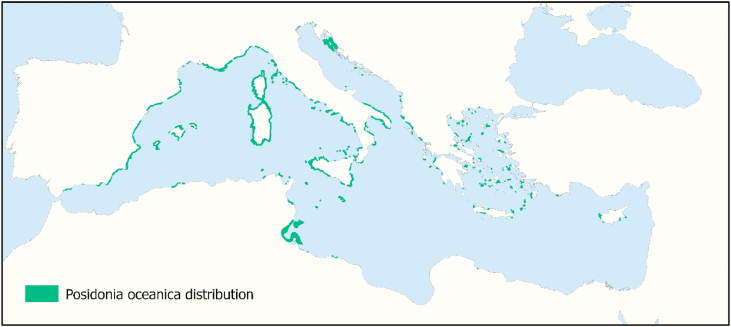
Distribution of Posidonia oceanica in the Mediterranean Sea. Data from UNEP-WCMC and Short (2021)*.*

Seagrass distribution data was taken from the seventh version of the Global Distribution of Seagrasses dataset (UNEP-WCMC and Short, 2021), which is made up of multiple local and regional data sources. The *P. oceanica* distribution in the Mediterranean Sea comes specifically from EMODnet Seabed Habitat data from Italy, France, and Greece (EMODnet Seabed Habitats, 2019), from the MEDISEH project for Malta (Borg et al., 2004; Borg and Schembri, 2002; Micallef et al., 2013; Scott Wilson Ltd ADI Associates, 2008), in addition to wider studies of the whole region (Procaccini and Mazzella, 1996; Telesca et al., 2015). All polygons that had *Posidonia oceanica* noted within them were considered relevant for this study, the final distribution dataset can be seen in Fig. 2.

For the MESA analysis, seagrass needs to be divided into grid cells that each represent a distinct environmental asset. Grids were computed using the standard MESA workflow as described by NEA and UNEP-WCMC (2020). Based on the Quarter Degree Grid Cell Standard used by MESA, which encompasses seven different levels ranging from 55 × 55 km (Level 1) to 850 × 850m (Level 7) (NEA and UNEP-WCMC, 20202), we chose level 3 grid cells for all visualizations in the following sections, which have a size of 0.125° × 0.125° and were therefore deemed most aligned to the temperature data described below.

The data described in Table 2 was downloaded for May to September to isolate summer periods which pose a higher heat stress on *P. oceanica* than winter heatwaves. The reduced-emissions scenario Representative Concentration Pathway (RCP) 4.5 (IPCC, 2014) was used as a basis for this model, in which carbon emissions continue to rise until 2040 and then drop off, to identify where seagrass is most likely to face risks under the most likely climate change scenario associated with severe emissions reductions. Given that average yearly maximum SST across the Mediterranean over a 30-year period has occurred in August at 24.3 °C (Sakalli, 2017) marine heatwaves were defined as the occurrence of SST >24 °C for at least 5 consecutive days, in line with a regional adaptation of the global definition of marine heatwaves by Oliver et al. (2021).

**Table 2:** Specifications of water temperature data used.

Data source	Copernicus Climate Change Service (Hall et al., 2019)
Model used	Proudman Oceanographic Laboratory Coastal Ocean Modelling System, European Regional Seas Ecosystem Model (POLCOMS-ERSEM)
Variable	Daily sea water potential temperature across the water column in Kelvin
Spatial resolution	0.1° × 0.1°
Representative Concentration Pathway	4.5

Raster files of daily potential sea temperature projections for the years 2046–2055 were considered to average around the year 2050. The raster files were stacked to produce a seasonal layer for the entire Mediterranean Sea, before later restricting it to only pixels that include *Posidonia oceanica*. All pixels that include *Posidonia oceanica* were considered, regardless of percentage coverage, and no buffer was applied.

The following information was extracted for each pixel of 0.1° × 0.1° (approx. 11 km):

• max SST per year,
• the longest duration of a MHW per year, defined as the maximum number of consecutive days that SST exceeded 24 °C,
• the number of heatwaves in a year, defined as the number of times a pixel had a temperature above 24 °C that lasts for a minimum of five consecutive days, and
• the time between heatwaves, defined as the maximum number of days where temperatures are below 24 °C, between periods of time of above 24 °C that lasted at least 5 days.

Each of the above criteria were considered independently and scored according to Table 1. The two impact severity results where averaged, as were the results for the potential for recovery. For each seagrass patch, these were combined to provide a single susceptibility score using the MESA susceptibility matrix in Table 3 (NEA and UNEP-WCMC, 2020).

**Table 3:** Susceptibility matrix to combine Impact Severity and Potential for Recovery scores (adapted from NEA and UNEP-WCMC, 2020).

Potential for recovery	1	1	2	2	3	3
	**2**	2	2	3	3	4
	**3**	2	3	3	4	4
	**4**	3	3	4	4	5
	**5**	3	4	4	5	5
		**1**	**2**	**3**	**4**	**5**
		**Impact Severity**				

### 2.2 Assessing the importance

The importance of seagrass patches was considered in relation to the provision of two key ecosystem services within which they are associated, namely reduction of coastal hazards and fisheries (Jackson et al., 2015; Satta et al., 2017). The aim was to distinguish areas where a decline in seagrass may pose a particularly high risk of socio-economic impacts relative to the region as a whole, and highlight countries that may be priorities for policy responses. Each seagrass patch was scored for importance between 1 (very low) and 5 (very high).

**Coastal hazards:** The relative risk to coastal communities from coastal hazards, specifically erosion and flooding, was assessed using the Coastal Risk Index (CRI) developed by Satta et al. (2017). The CRI combines thirteen variables covering both physical and socio-economic dimensions of coastal forcing, coastal vulnerability, and coastal exposure to assign levels of risk from coastal hazards to Mediterranean coastlines.

Seagrass has been shown to contribute to protecting coastal areas from a variety of coastal hazards by changing hydrodynamic conditions. Multiple studies have shown that nearshore seagrass beds reduce wave speed and height, as well as erosion by storing and capturing sediments (Bradley and Houser, 2009; Peterson et al., 2002; Spalding et al., 2014). In the face of increased SST, the ability of seagrass to provide this service is likely to decline (Ondiviela et al., 2014).

As provision of this service declines, it will be important for decision-makers to implement alternative solutions for the management of coastal hazards. Therefore, in this case study, the percentage of the country's coastline assessed as ‘very high’ risk was applied as the importance criteria (Satta et al., 2017) (Table 4), to highlight areas where policy changes may be most needed.

**Table 4:** Importance scores given to EEZ based on the % of coastline assessed as very high risk (for coastal hazards), and the % of regional capture production of seagrass dependent species (for fisheries).

Importance Score	Percentage of country coastline at very high risk from coastal hazards	Percentage of seagrass-dependent fish caught within EEZ
1 (Very low)	<5%	<5%
3 (Moderate)	>5%, <20%	>5%, <20%
5 (Very high)	>20%	>20%

**Fisheries:** Seagrass-dependent fisheries were identified using the “seagrass residency index” (SRI) (Jackson et al., 2015) which scores the dependency of Mediterranean fish species, based on the weighted sum of averages of the estimated residence time in seagrass compared to other habitats at each life stage. We considered the six commercial taxa with the highest SRI to map seagrass-dependent fisheries in the Mediterranean region as an indicator for the potential dependency on *Posidonia oceanica*. These were Axillary Seabream (*Pagellus acarne*), Common pandora (*Pagellus erythurus*), Poor cod (*Trisopetrus minutus*), Caramote shrimp (*Melicertus kerathurus*), Atlantic scallop (*Pecten maximus*), and Scorpionfish and rockfish (Scorpaena). Given the breadth of Scorpaena as a taxa group, the red scorpionfish (*Scorpaena scrofa*), Black scorpion fish (*Scorpaena porcus*), and the Small Red Scorpionfish (*Scorpaena notata*) were used to represent Scorpaena within the analysis.

To determine the importance of each country to the total regional catch of these seagrass-dependent species, capture production statistics were obtained from the United Nations Food and Agriculture Organization (FAO) General Fisheries Commission for the Mediterranean (GCFM) Capture Production dataset (FAO, 2021). The total regional catch of each of the six taxa was averaged between 2014 and 2018. The average values were then used to calculate the percentage of total regional catch occurring in each exclusive economic zone (EEZ), as an indicator of which countries contribute most to regional seagrass-dependent fisheries production.

#### 2.2.1 Data analysis

To determine importance at a national scale, the EEZ percentages for both the proportion of coastline assessed as very high hazard risk, and the proportion of regional capture production of seagrass-dependent species were converted into importance scores using defined thresholds (Table 4). These thresholds were based on the distribution of the country percentages, and help to identify ‘hotspot’ areas for each importance criteria. As the available importance data is less granular than the susceptibility data, only three instead of five different thresholds were chosen to avoid overemphasizing artificial distinctions between thresholds. These scores where then applied to all seagrass patches that occur within the respective EEZ.

### 2.3 Calculating sensitivity

**Fig. 3:**
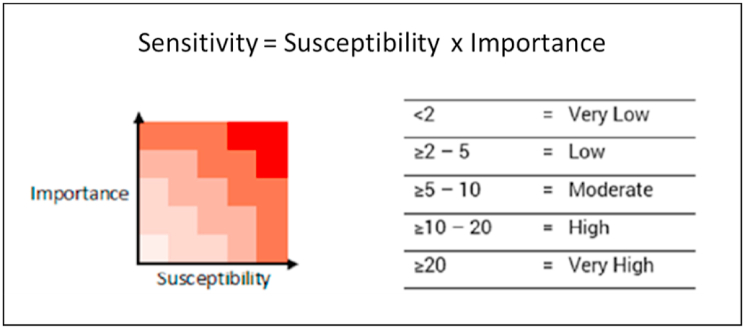
MESA sensitivity matrix (NEA and UNEP-WCMC, 2020).

Susceptibility and importance scores were then multiplied for each seagrass patch, using the MESA sensitivity matrix (Fig. 3) to provide an overall sensitivity from Very Low to Very High (NEA and UNEP-WCMC, 2020).

## 3 Results

**Fig. 4:**
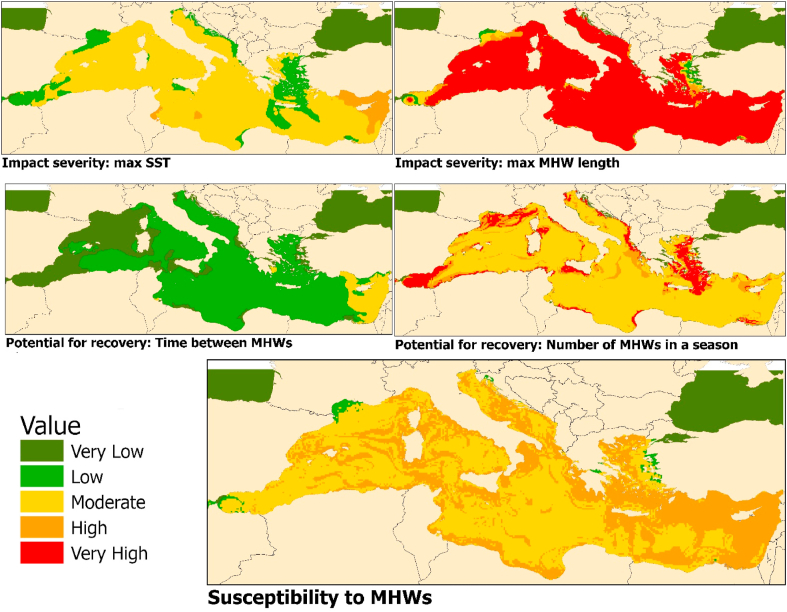
Results from the impact severity and potential for recovery analysis, and the resulting overall susceptibility scores.

Fig. 4 shows the five resulting maps of the susceptibility analysis. While no area in the Mediterranean will reach a daily maximum sea surface temperature of above 32 °C, there is also no area that will not at least reach temperatures between 24 and 27 °C, with most areas reaching above 27 or even 29 °C. Almost all areas of the Mediterranean will experience heatwaves that last longer than a month, which is long enough to cause long-term damage to seagrass. Most areas will experience between 2 and 4 distinct heatwave periods per season, with between 40 and 60 days between heatwaves at non-heat stress temperatures, allowing seagrass meadows to recover. The Eastern part of the Mediterranean around Cyprus, as well as the Tunisian coast will experience the hottest Sea Surface Temperatures, combined with only short periods of time in between heatwaves.

Overall, no areas of the Mediterranean will experience “very low” or “very high” susceptibility of seagrass to MHWs by mid-century. The largest parts will experience moderate susceptibility, while streaks around the coasts, where the majority of seagrass is distributed, will experience high susceptibility to MHWs. Only small pockets in the South of France and by the coast of Turkey willhave low susceptibility of seagrass to MHWs.

Disclaimer: The designations employed and the presentation of material on this map do not imply the expression of any opinion whatsoever on the part of the Secretariat of the United Nations concerning the legal status of any country, territory, city or area or of its authorities, or concerning the delimitation of its frontiers or boundaries.

### 3.1 Coastal hazards

**Fig. 5:**
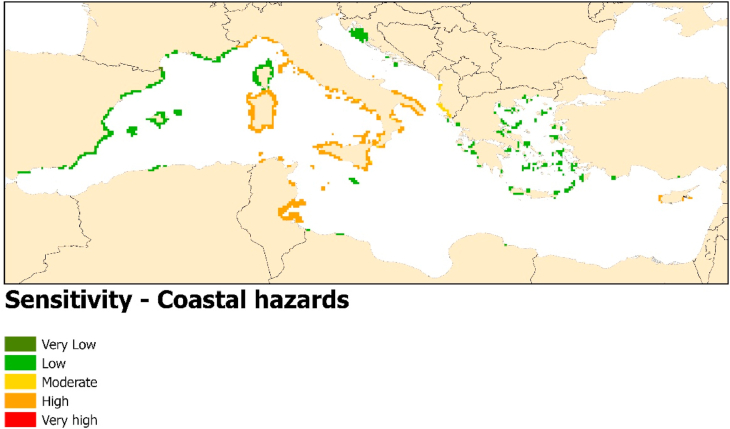
Environmental sensitivity results based on coastal hazards importance criteria.

With relation to the management of coastal hazards, the loss of ecosystem services as a result of the predicted impacts of MHWs on *P. oceanica* are likely to be felt most severely in the central Mediterranean in the coastal regions of Italy and Tunisia, as well as in Cyprus (Fig. 5). All other countries only show a low sensitivity to the loss of this ecosystem service, with just Albania showing a moderate sensitivity.

Disclaimer: The designations employed and the presentation of material on this map do not imply the expression of any opinion whatsoever on the part of the Secretariat of the United Nations concerning the legal status of any country, territory, city or area or of its authorities, or concerning the delimitation of its frontiers or boundaries.

### 3.2 Fisheries

**Fig. 6:**
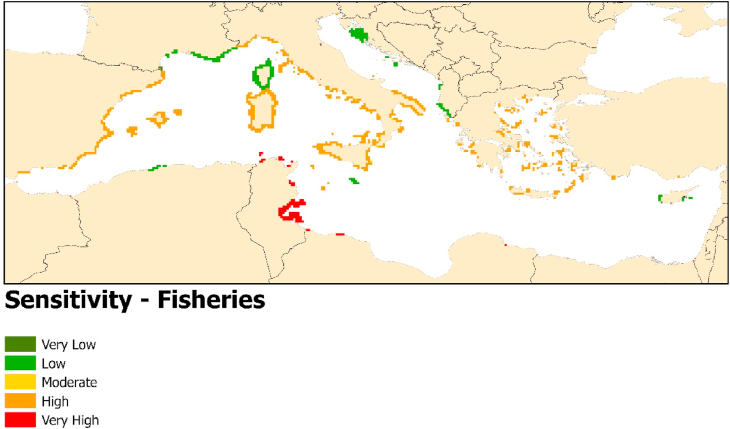
Environmental sensitivity to loss of fisheries provisioning as a result of MHW-induced seagrass loss.

Fig. 6 shows that the loss of seagrass-dependent fisheries is likely to be most severely felt in the coastal regions of Tunisia and Libya where sensitivity is Very High due to high reliance on seagrass-dependent fish species, as well as high susceptibility of seagrass to MHWs. Greece, Italy, Turkey, and Spain show high sensitivity, while all other countries around the Mediterranean have a low sensitivity to seagrass-dependent fishery loss.

Disclaimer: The designations employed and the presentation of material on this map do not imply the expression of any opinion whatsoever on the part of the Secretariat of the United Nations concerning the legal status of any country, territory, city or area or of its authorities, or concerning the delimitation of its frontiers or boundaries.

## 4 Discussion

This study should be understood as a prioritization exercise for further action that is trialling a ready-to-use method that policymakers in low-capacity regions can easily apply themselves, with limited computing power. In this paper, we have demonstrated the ability of the MESA methodology to provide rapid, high-level assessments of sensitivity to climate change. While response to climate change is complex and context-dependent, the categorical approach allows decision-makers to identify areas where impacts are likely to be greatest and prioritize these for in-depth study. As all risk analyses, this approach has inherent uncertainty, and all thresholds should be seen as approximate values between which risk is very likely to increase. The speed at which environmental sensitivity mapping can be deployed enables it to play an increasingly important role in the policy-making process, as the effects of climate change intensify and the need to direct finite resources increases.

The results of this study are a combination of reliance on ecosystem services, as well as the susceptibility of seagrass beds to degradation through increasing MHW intensity and frequency. These factors need to be looked at in combination to adequately adapt to climate change impacts, and to find nuanced solutions to a complex problem. We have demonstrated that the implications of MHW could be significant under the conservative RCP 4.5, while current trends indicate we will exceed this pathway (Schwalm et al., 2020). In addition, given the uncertainty about which pathway is most likely, regional policies for the Mediterranean must consider the potential implications of heatwave-induced seagrass loss up to and including the RCP 8.5 and plan accordingly (Schwalm et al., 2020), which would likely push most areas to a high or very-high sensitivity according to this methodology.

Countries showing a very high or high sensitivity towards loss of ecosystem services should be prioritized for more in depth study of the role that seagrass beds play in coastal protection and fish stocks. Especially in the vicinity of heavily populated areas, the implications of reduced coastal protection are likely to be felt most acutely. These regions may need to shift their reliance to alternative methods of mitigating coastal hazards, for example through other nature-based solutions such as the restoration of wetlands that are more adapted to future temperatures in the Mediterranean region, or through investment in grey or green infrastructure. Lower-income countries with high sensitivity, such as Tunisia, should be especially forward-thinking to adapt to the impact of climate change.

Previous work has also highlighted the economic value of seagrass to Tunisian fisheries, and how direct pressures from industrial pollution caused declines in fisheries value (el Zrelli et al., 2020). Cumulative impacts of heatwaves and low water quality have been shown to reduce the ability of seagrass to resist or recover from further stress (Collier and Waycott, 2014). Further study should therefore be conducted to understand whether the mitigation of other pressures, such as industrial pollution, could potentially improve the resilience of seagrass beds in the face of increased MHWs. Where this is not feasible, larger-scale policy changes may be needed to support coastal communities to diversify economic opportunities to other fishery and non-fishery activities.

Adaptation to these problems should also incorporate efforts to avoid further loss of seagrass by addressing other pressures. As well as increasing coverage of Marine Protected Areas, focus should be on flexible management plans that enable rapid adaptation to the effects of environmental variation, such as heatwaves (Holbrook et al., 2020). It will however also incorporate investment to support coastal communities to adapt to the impacts of climate change, ensuring these communities are protected from the physical implication, and supported in their efforts to achieve economic security.

## 5 Conclusions

This paper demonstrates the potential risk to communities within the Mediterranean due to the loss of ecosystem services brought about by the impact of increasing MHW on *P. oceanica* seagrass beds. It highlights the continued need to support global efforts for rapid and sustained reductions in global carbon emissions, as well as adaptation to the complex effects climate change might have on local economies and communities. Countries with very high to high sensitivity to either ecosystem service should reduce additional pressures to seagrass, and should aim to diversify fisheries and coastal protection efforts. Especially lower-income countries need to be prepared to combat additional coastal hazards or loss of food sources. MESA is an easily useable sensitivity mapping method that can be applied by low-capacity stakeholders and policymakers to further refine sensitivity maps within countries to a smaller scale.

## Funding information

This research was funded by European Union's Horizon 2020 Research and Innovation Program (H2020-BG-12-2016-2), grant number No. 727277—ODYSSEA (Towards an integrated Mediterranean Sea Observing System). The article reflects only authors' views, and the Commission is not responsible for any use that may be made of the information it contains. The funders had no involvement in study design or implementation.

## CRediT authorship contribution statement

**Sara Pruckner:** Conceptualization, Data curation, Formal analysis, Investigation, Methodology, Resources, Validation, Visualization, Writing – original draft, Writing – review & editing. **Jacob Bedford:** Formal analysis, Methodology, Writing – original draft. **Leo Murphy:** Conceptualization, Methodology, Software, Validation, Writing – original draft. **Joseph A. Turner:** Conceptualization, Formal analysis, Investigation, Methodology, Validation. **Juliet Mills:** Conceptualization, Data curation, Writing – original draft.

## Declaration of competing interest

The authors declare that they have no known competing financial interests or personal relationships that could have appeared to influence the work reported in this paper.

## Data Availability

All code and resulting spatial data are publicly available via GitHub at: https://github.com/sarapruck/MESA-MHW-Poceanica.git.
